# Review of Artificial Intelligence for Automated Assessment of Lines, Drains, and Airways on Pediatric and Adult Chest Radiographs

**DOI:** 10.3390/pediatric18050119

**Published:** 2026-09-11

**Authors:** Junqi Wang, Lili He, Gary R. Schooler, Alexander J. Towbin, Hailong Li

**Affiliations:** 1 Imaging Research Center, Cincinnati Children’s Hospital Medical Center, Cincinnati, OH 45229, USA; junqi.wang@cchmc.org (J.W.); lili.he@cchmc.org (L.H.); 2 Department of Radiology, Cincinnati Children’s Hospital Medical Center, Cincinnati, OH 45229, USA; gary.schooler@cchmc.org (G.R.S.); alexander.towbin@cchmc.org (A.J.T.); 3 Artificial Intelligence Imaging Research Center, Cincinnati Children’s Hospital Medical Center, Cincinnati, OH 45229, USA; 4 Computer Science, Biomedical Engineering, Biomedical Informatics, University of Cincinnati College of Medicine, Cincinnati, OH 45229, USA; 5 Department of Radiology, University of Cincinnati College of Medicine, Cincinnati, OH 45229, USA

**Keywords:** medical device, chest radiography, AI, ETT, segmentation, LDA, central venous catheters, nasogastric tube

## Abstract

The accurate placement of lines, drains, and airways (LDAs) is essential for the safe management of critically ill patients, as malpositioned medical devices can result in severe complications ranging from ineffective treatment to life-threatening injury. Chest radiography (CXR) is the primary imaging modality for confirming the placement of many LDAs; however, the growing volume and complexity of bedside CXRs have created increasing demand for rapid, reliable interpretation. Recent advances in artificial intelligence (AI) have enabled automated detection, localization, and position assessment of LDA devices, supporting opportunities to improve clinical workflow and patient safety. This review summarizes recent developments in AI algorithms for automated LDA assessment on CXRs, emphasizing their clinical applications, technical approaches, performance, and limitations. The review begins with clinical characteristics, radiographic appearance, and placement criteria of common LDA categories, followed by a survey of AI methods for presence detection, device localization, and position classification. Although some models have achieved performance approaching that of radiologists, most do not perform at the level needed for clinical deployment. Future research should prioritize multicenter validation, standardized annotation protocols, pediatric-specific datasets, and anatomically informed models capable of simultaneously evaluating multiple LDA devices. Advances in these areas will facilitate the integration of AI-assisted LDA assessment into clinical decision support and ultimately improve patient care.

## 1. Introduction

Lines, drains, and airways (LDAs) are essential indwelling medical devices used for respiratory support, vascular access, enteral nutrition, gastrointestinal decompression, hemodynamic monitoring, and other therapeutic interventions [1,2,3]. Because these devices may be malpositioned during insertion or subsequently displaced by patient movement, routine care, or procedural manipulation, accurate assessment of their position is essential to prevent complications and guide clinical management [4,5,6].

Chest radiography (CXR) is the primary imaging modality used to confirm placement and monitor many of these devices [7,8,9,10]. However, interpreting bedside CXRs can be challenging, particularly when multiple LDAs overlap with one another. Portable radiographs are frequently obtained under technically suboptimal conditions, with supine positioning, reduced image quality, low contrast, and altered anatomy making these thin medical devices difficult to identify accurately. These technical challenges are compounded by increasing imaging volumes, radiologist workforce shortages, and competing demands from cross-sectional imaging, emergency examinations, and other high-priority studies, which may delay interpretation of bedside CXRs by several hours in busy clinical settings [11,12,13,14,15,16].

To address these challenges, artificial intelligence (AI) has emerged as a promising approach for automated LDA assessment on CXRs. AI can automatically detect LDA devices, localize device tips, and identify potential malposition, supporting more efficient image interpretation [13,17,18]. For the foreseeable future, AI is intended to function as a clinical decision support (CDS) tool rather than replace clinical judgment. By automatically identifying LDA devices and potential malposition, AI may improve workflow efficiency and reporting consistency [14,19,20]. AI may also improve access to timely imaging support in hospitals with radiologist shortages or limited subspecialty expertise by providing preliminary assessment when expert interpretation is not immediately available [14,21].

An earlier review by Yi et al. [22] summarized computer-aided approaches for catheter and tube assessment on radiographs and highlighted key challenges in automated device evaluation; since then, substantial advances in AI-based LDA assessment have been reported across a broader range of devices and clinical applications. While automated assessment of LDAs has advanced rapidly, the literature remains fragmented across individual devices, algorithms, and clinical applications. This review provides a comprehensive overview of AI methods for automated LDA assessment on CXRs in both adult and pediatric populations. We organize the AI applications into three core tasks: presence detection, device localization, and position classification. For each task, we compare performance across multiple LDA categories and discuss the key challenges and future directions for clinical translation.

## 2. Methodology

### Search Strategy

**Figure 1:**
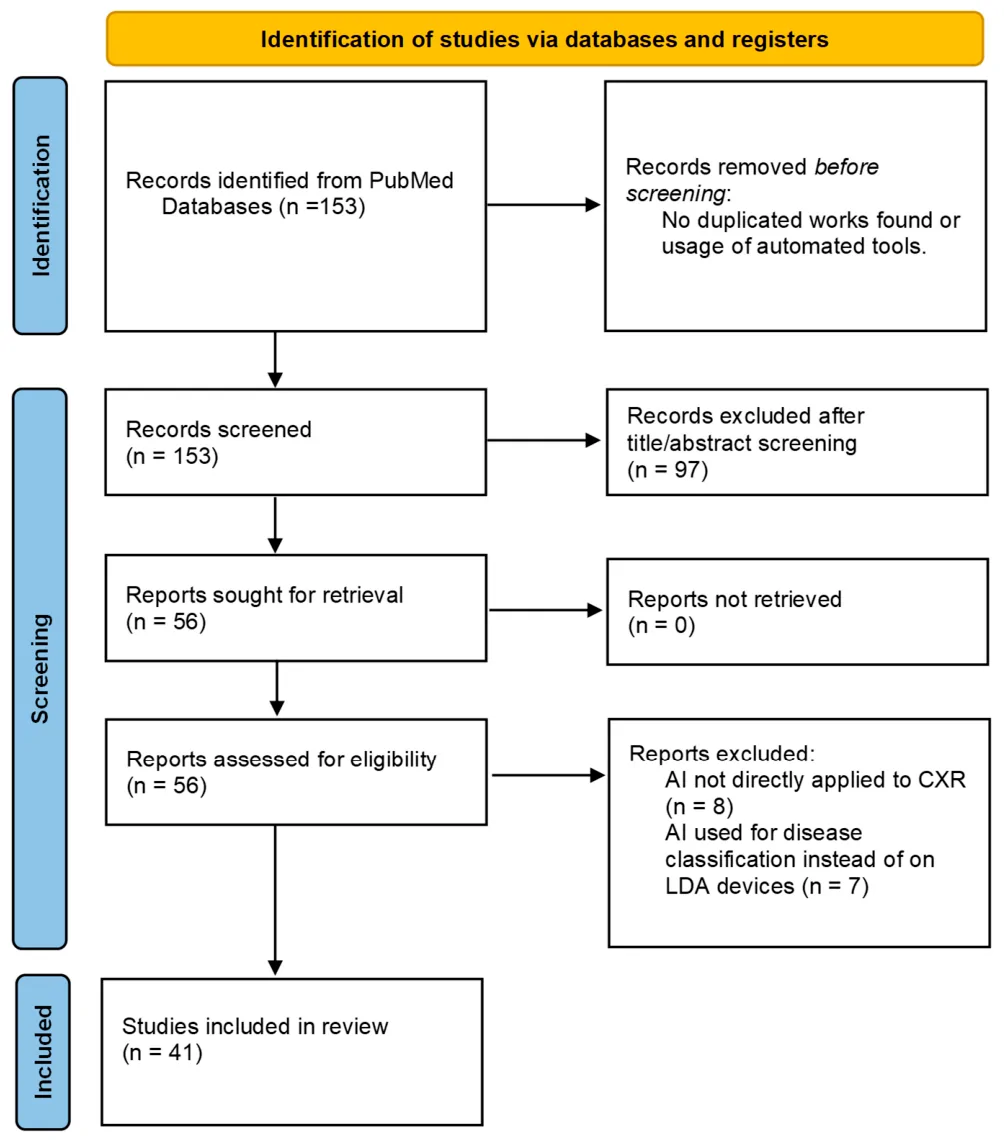
Flow diagram of the literature search and screening procedure.

To promote transparency and reproducibility, a structured literature search strategy was implemented, and the selection process was documented using a PRISMA-style (Preferred Reporting Items for Systematic Reviews and Meta-Analyses) flow diagram [23]. The literature search was conducted in PubMed and limited to a 10-year publication period (13 June 2016, to 13 June 2026) to capture recent advances in AI, especially deep learning, for LDA assessment (Figure 1). The LDA devices included in this review were selected a priori in consultation with board-certified radiologists and represent four major categories encompassing 16 devices routinely evaluated on chest radiographs. The search strategy was designed to capture studies evaluating AI models for LDA assessment in both adult and pediatric populations.

To identify relevant studies, we developed a comprehensive PubMed search strategy encompassing AI methodologies, LDA devices, and CXR in both adult and pediatric populations. The following Boolean query was applied: (“Deep Learning”[MeSH] OR “Artificial Intelligence”[MeSH] OR “Machine Learning”[MeSH] OR “Computer Vision”[tiab] OR “Neural Network*”[tiab] OR “Convolutional Neural Network*”[tiab] OR “CNN”[tiab] OR “Automated Detection”[tiab] OR “Object Detection”[tiab] OR “Image Segmentation”[tiab] OR “YOLO”[tiab] OR “U-Net”[tiab] OR “DeepLab*”[tiab] OR “Transfer Learning”[tiab] OR “Localization”[tiab] OR “Localisation”[tiab] OR “Automated Tracking”[tiab]) AND (“Endotracheal”[tiab] OR “ETT”[tiab] OR “Tracheostomy”[tiab] OR “Nasogastric”[tiab] OR “NGT”[tiab] OR “Orogastric”[tiab] OR “OGT”[tiab] OR “Nasoduodenal”[tiab] OR “NDT”[tiab] OR “Nasojejunal”[tiab] OR “NJT”[tiab] OR “Gastrojejunal”[tiab] OR “GJ tube”[tiab] OR “Gastrostomy”[tiab] OR “G tube”[tiab] OR “Replogle”[tiab] OR “PICC”[tiab] OR “CVC”[tiab] OR “Central Line*”[tiab] OR “Internal Jugular”[tiab] OR “Subclavian”[tiab] OR “UVC”[tiab] OR “UAC”[tiab] OR “Chest Tube*”[tiab] OR “Thoracostomy”[tiab] OR “Pleural Drain”[tiab] OR “Percutaneous Catheter Drainage”[tiab] OR “PCD”[tiab] OR “Umbilical Catheter*”[tiab] OR “Catheter*”[tiab] OR “Medical Tube*”[tiab] OR “Feeding tube*”[tiab] OR “Enteric tube*”[tiab] OR “Malposition*”[tiab]) AND (“Radiography, Thoracic”[MeSH] OR “Radiography, Abdominal”[MeSH] OR “Chest X-ray”[tiab] OR “CXR”[tiab] OR “Chest Radiograph*”[tiab] OR “Plain film”[tiab] OR “Digital Radiography”[tiab] OR “Infant, Newborn”[MeSH] OR “Pediatrics”[MeSH] OR neonat*[tiab] OR pediatric*[tiab] OR paediatric*[tiab] OR infant*[tiab] OR newborn*[tiab] OR NICU[tiab] OR “premature”[tiab] OR “preterm”[tiab]) AND (“Radiograph*”[tiab] OR “X-ray”[tiab] OR “Imaging”[tiab])).

The search was designed to identify peer-reviewed studies that developed, validated, or clinically evaluated AI algorithms for LDA assessment on CXRs, including image-level classification, full-course semantic segmentation, and key point detection.

Studies evaluating 3D imaging modalities (CT or MRI), non-imaging physiological verification (e.g., ECG guidance), or manual ultrasound tracking without an automated computer vision component were excluded.

The search identified 153 records. The literature screening was conducted by the authors (JW and HL) in two sequential phases. First, the authors screened the titles and abstracts of all retrieved records against the eligibility criteria, excluding articles that clearly did not meet inclusion requirements. Second, for any records where eligibility could not be determined from the title and abstract, the authors reviewed the full text to make a final inclusion decision. After manual screening, 41 studies met the eligibility criteria and were included in the final review.

## 3. Clinical Background for LDA Devices

This section summarizes the clinical applications, proper placement criteria, and potential complications of the four primary categories of LDA devices.

**Figure 2:**
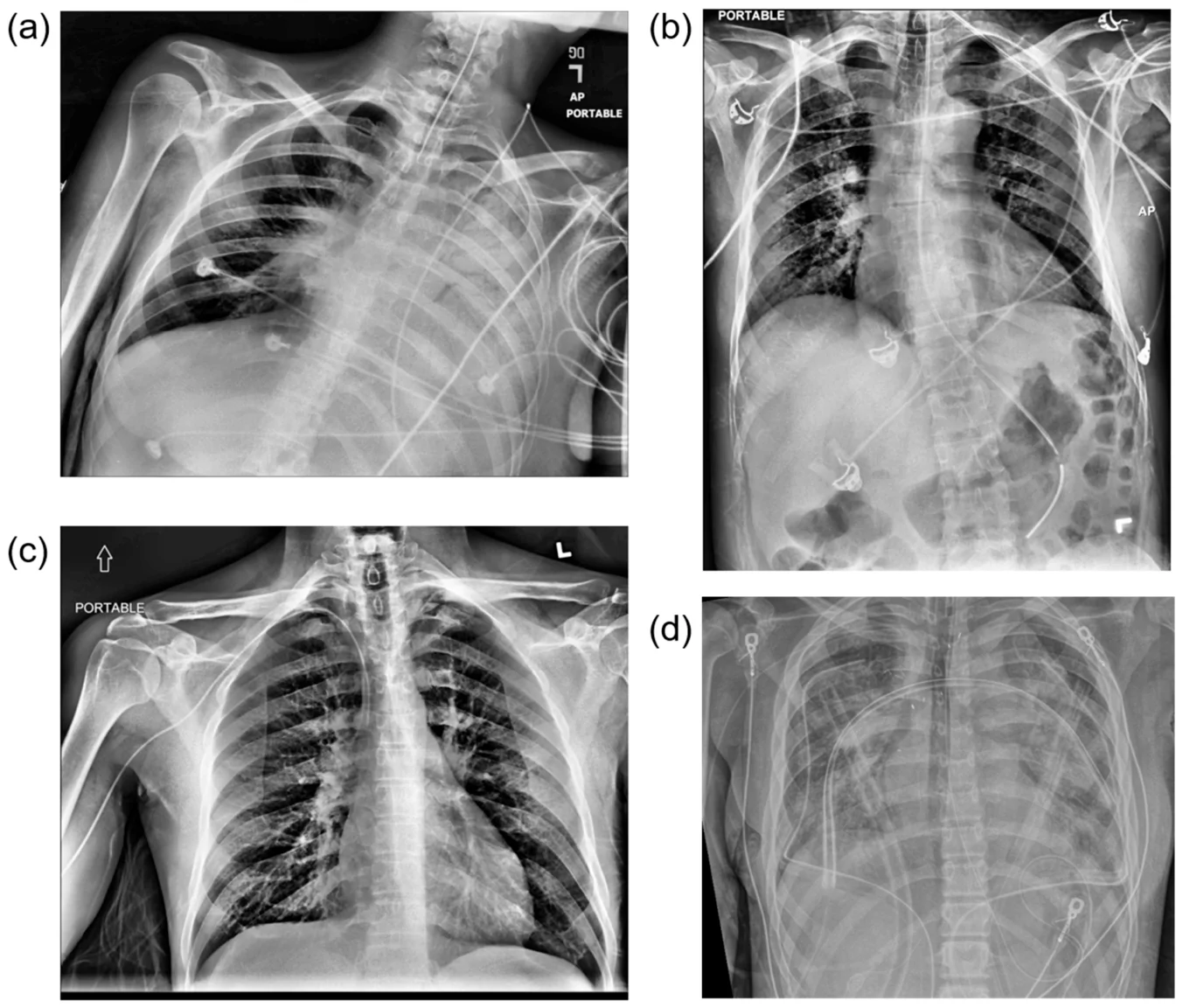
Chest radiographs demonstrating the four primary categories of line, drain, and airway (LDA) devices. Images have been contrast-enhanced to improve visualization of device trajectories and distal tips. (**a**) Artificial Airway: Frontal chest radiograph demonstrating an endotracheal tube (ETT) with the tip in the mid-trachea above the carina. (**b**) Enteric Tube: Frontal chest radiograph demonstrating a nasogastric tube (NGT) coursing into the stomach with the tip in the mid-stomach. (**c**) Central Venous Catheter: Frontal chest radiograph demonstrating a peripherally inserted central catheter (PICC) with the tip in the lower third of the superior vena cava. (**d**) Chest Tube: Frontal chest radiograph demonstrating four chest tubes, two within each hemithorax.

Figure 2 illustrates four primary categories of LDA device on CXRs.

### 3.1. Artificial Airways

Artificial airways are tubes inserted into the respiratory tract to maintain airway patency, protect the airway, and facilitate mechanical ventilation. This category primarily comprises two devices:

Endotracheal Tube (ETT): An ETT is an airway tube inserted through the mouth or nose into the trachea to maintain a patent airway and facilitate mechanical ventilation. Proper ETT placement requires the distal tip to lie in the mid-trachea, between the thoracic inlet and the carina [24,25]. A low-positioned tube may enter a mainstem bronchus, resulting in unilateral lung hyperinflation, contralateral atelectasis, hypoxemia, or pneumothorax. Conversely, positioning the tube too high can result in laryngeal spasm, accidental extubation, and vocal cord damage [26,27].

Tracheostomy Tube: A tracheostomy tube is a short, curved airway device inserted directly into the trachea through a surgical opening (stoma) in the anterior neck. It provides long-term respiratory support. Proper placement of the tracheostomy tube requires the distal tip to be centrally aligned within the tracheal lumen, terminating approximately midway between the stoma site and the carina. Misplacement or angulation can cause tracheal wall erosion, subcutaneous emphysema, or accidental decannulation into the pretracheal space [9].

### 3.2. Enteric Tubes

Enteric tubes are flexible catheters inserted into the gastrointestinal tract to provide nutrition, administer medications, or decompress the stomach and bowel.

Nasogastric Tube (NGT): An NGT is a feeding and decompression tube inserted through the nose that traverses the esophagus to terminate in the stomach. It is used for short-term enteral nutrition, medical administration, gastric decompression, and removal of gastric contents. Proper NGT placement requires the tube to remain in the midline of the chest, cross below the diaphragm, and terminate within the stomach, with the side-port distal to the gastroesophageal junction [28]. Inadvertent placement in the respiratory tract is the primary clinical risk, leading to associated pulmonary complications [28].

Orogastric Tube (OGT): An OGT functions identically to an NGT but is inserted through the mouth to terminate in the stomach. It is commonly used in intubated adults and neonates to avoid nasal airway obstruction. It follows the same placement criteria as an NGT, and incorrect placement similarly risks accidental airway insertion or esophageal perforation [29].

Nasoduodenal Tube (NDT): An NDT is a thin feeding tube inserted nasally that passes through the stomach and pylorus to terminate in the duodenum. It is used to provide post-pyloric enteral nutrition. Proper placement requires the tip to extend beyond the pylorus within the C-loop of the duodenum, avoiding looping or coiling within the gastric fundus. Inadvertent misplacement can result in the tube coiling in the esophagus or entering the respiratory tract, which may lead to severe complications such as lung or pleural cavity perforation [29].

Nasojejunal Tube (NJT): An NJT is an elongated feeding tube inserted through the nose and advanced through the stomach and duodenum to terminate at or beyond the ligament of Treitz. It provides long-term post-pyloric nutrition while minimizing the risk of aspiration. Proper placement requires the tube to pass through the pylorus and advance beyond the ligament of Treitz into the proximal jejunum. Inadvertent misplacement carries the risk of entering the airway or pleural cavity instead of the small bowel, potentially causing significant pulmonary trauma [29].

Gastrostomy Tube (G-Tube): A G-tube is a short, direct access catheter inserted through an abdominal incision (stoma) into the stomach to provide direct gastric access for long-term nutrition. Proper placement relies on the internal bolster or water-filled balloon being securely anchored against the gastric wall. Dislodgement into the peritoneal space can cause peritonitis [30]. Tubes positioned within the anterior abdominal wall can cause cellulitis.

Gastrojejunal Tube (GJ-Tube): A GJ-tube is a dual-port device inserted directly through the abdominal wall into the stomach. The tube has an extended jejunal limb that terminates at or beyond the ligament of Treitz. The tube allows for simultaneous gastric access/decompression and direct jejunal feeding. Proper placement requires the gastric retention balloon to be flush against the anterior stomach wall. The jejunal limb should extend beyond the pylorus into the duodenum or jejunum without kinking. Misplacement of a gastrojejunal tube can cause feeding intolerance, vomiting, increased pulmonary aspiration risk, and small bowel intussusception and ischemic necrosis [30].

Replogle Tube: A Replogle tube is a dual-lumen suction catheter inserted through the nose or mouth for continuous decompression of the upper gastrointestinal tract. In most patients, the tip terminates within the stomach. In neonates with esophageal atresia, however, it is intentionally positioned within the blind-ending proximal esophageal pouch to continuously aspirate secretions and reduce the risk of aspiration. Appropriate placement depends on the clinical indication [31].

### 3.3. Central Venous Catheters (CVCs)

Central venous catheters (CVCs) are vascular access devices inserted into large systemic veins to administer medications, deliver total parenteral nutrition, obtain blood samples, and monitor central hemodynamic status. This group includes:

Internal Jugular (IJ) Line: An IJ line is a central catheter inserted directly into the internal jugular vein in the neck that courses inferiorly through the neck and terminates in the lower superior vena cava (SVC), near the cavoatrial junction [32]. Over-insertion of the line into the right atrium increases the risk of cardiac arrhythmias, atrial perforation, and cardiac tamponade, whereas under-insertion increases the risk of venous thrombosis and chemical phlebitis [18,33,34].

Subclavian Line: A subclavian line is a central catheter inserted beneath the clavicle into the subclavian vein, coursing medially into the brachiocephalic vein and terminating in the lower SVC, near the cavoatrial junction [32]. Excessive advancement of the catheter into the right atrium heightens the risk of cardiac arrhythmias, atrial perforation, and tamponade, while inadequate insertion increases the risk of local venous thrombosis and chemical phlebitis.

Peripherally Inserted Central Catheter (PICC): A PICC is a long central venous catheter inserted through a peripheral vein to provide prolonged intravenous access for medications, parenteral nutrition, chemotherapy, blood products, and other intravenous therapies. The catheter may be inserted through veins of either the upper or lower extremity. Upper-extremity PICCs typically terminate at the cavoatrial junction or within the lower SVC, whereas lower-extremity PICCs terminate at the inferior cavoatrial junction or within the upper inferior vena cava (IVC) [18,33,35]. Advancing a PICC tip into the right atrium heightens the risk of arrhythmias, atrial perforation, and tamponade, while under-advancing it, leaving the tip within peripheral or axillary veins, increases the risk of localized thrombosis and chemical phlebitis.

Two specialized vascular catheters are commonly used in neonatal intensive care units through umbilical access.

Umbilical Venous Catheter (UVC): A UVC is a neonatal catheter inserted through the cut umbilical vein that travels superiorly through the ductus venosus to terminate in the IVC, right at its junction with the right atrium. Proper placement requires the tip to sit at or just above the diaphragm. Low positioning in the portal vein risks thrombosis and hepatic parenchymal infiltration, while high positioning in the heart risks arrhythmia [32].

Umbilical Arterial Catheter (UAC): A UAC is a neonatal catheter inserted through an umbilical artery that travels inferiorly into the internal iliac artery before coursing superiorly into the aorta. It allows continuous blood pressure monitoring and arterial blood gas sampling. Proper placement requires the tip to terminate either in a high position (above the celiac axis) or a low position (below the inferior mesenteric artery) to avoid major aortic branches and reduce the risk of visceral infarction or arterial thrombosis [36].

### 3.4. Drains

Drains are flexible catheters inserted into the body to evacuate fluid or gas collections and restore normal physiology. In the thorax, they function to restore negative intrapleural pressure, relieve mass effect on the mediastinum, and facilitate complete lung re-expansion [14,37]. This group primarily includes two categories of drainage systems:

Thoracic Drains (Chest Tubes): A thoracic drain is a large-bore or small-bore rigid plastic tube inserted to evacuate air or fluid from the pleural space or mediastinum. Most thoracic drains are inserted through an intercostal approach into the pleural cavity, although mediastinal drains are commonly placed through a subxiphoid approach following cardiothoracic surgery. For pleural drains, all drainage side holes (fenestrations) should lie completely within the pleural space, with the tip directed apically for pneumothorax or basally for pleural effusions. Malposition may result in inadequate drainage, pain, or injury to adjacent structures [37].

Percutaneous Catheter Drainage (PCD): A PCD is a small-bore, flexible drainage catheter, typically with a rounded pigtail configuration, that is inserted under image guidance (e.g., ultrasound or CT) to evacuate pleural or other fluid collections. Pigtail catheters are commonly used for pleural effusions of varying sizes, depending on the clinical indication and operator preference. Proper placement requires the pigtail locking mechanism to be fully deployed within the target collection. The catheter should remain free of kinking or migration. Malpositioning or kinking of these narrow-lumen catheters leads to obstruction and impaired drainage [37].

## 4. AI Tasks and Architectures for Automated LDA Assessment

**Figure 3:**
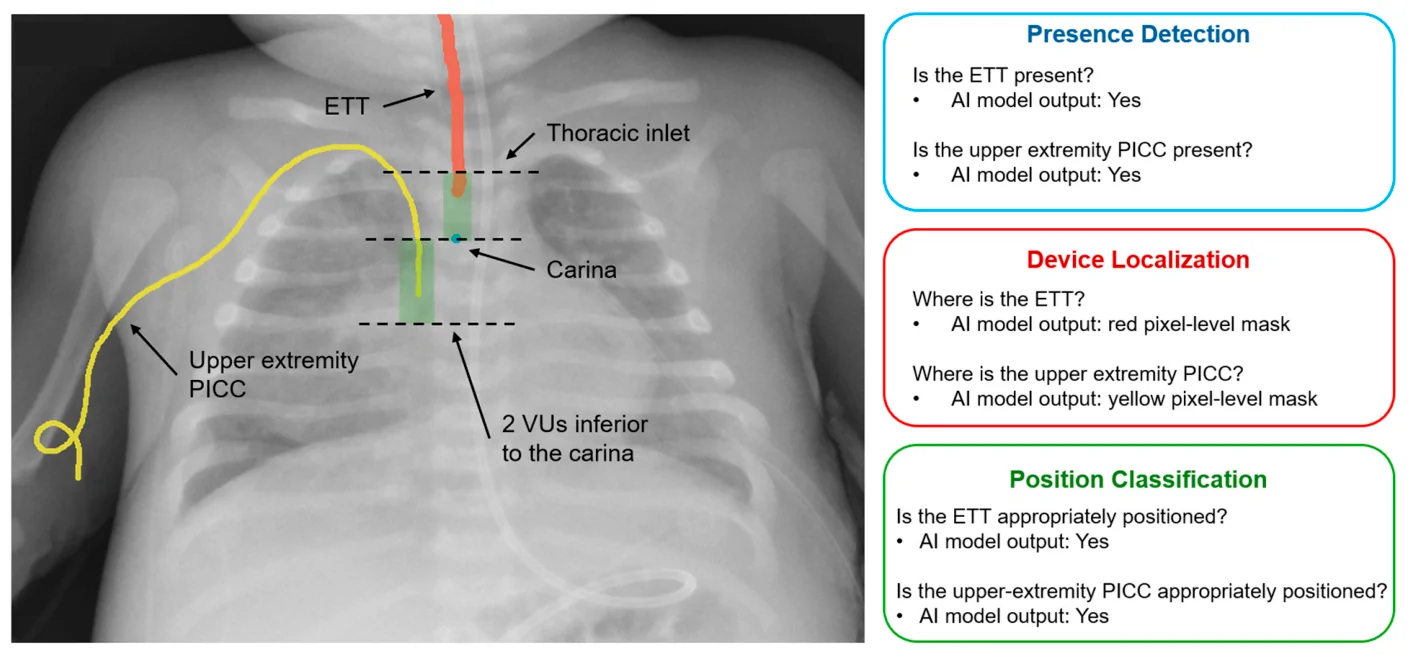
Conceptual overview of three AI tasks applied to LDA assessment on chest radiography. Representative neonatal chest radiograph demonstrating three common AI paradigms for LDA assessment: (1) Presence Detection: determining whether a targeted LDA is present (e.g., ETT present: yes; upper extremity PICC present: yes). Untargeted devices present on the radiograph (such as the enteric tube and chest tube) are not evaluated by the AI models. (2) Device Localization: segmenting the targeted LDA full course and/or identifying its distal tip (e.g., red pixel-level mask for ETT, yellow mask for upper extremity PICC). (3) Position Classification: determining whether the LDA distal tip is appropriately positioned relative to anatomical landmarks (e.g., Is the ETT appropriately positioned? Yes. Is the upper-extremity PICC appropriately positioned? Yes). ETT, endotracheal tube; LDAs, lines, drains, and airways; PICC, peripherally inserted central catheter; VU, vertebral unit.

AI applications for LDA assessment on CXRs can be broadly categorized into three tasks: presence detection, device localization, and position classification. These tasks represent progressively more complex stages of automated image analysis, from determining whether a device is present, to identifying its location, to assessing whether it satisfies established clinical placement criteria (Figure 3). A variety of AI architectures have been developed to accomplish these tasks, with deep learning approaches predominating in the current literature.

### 4.1. Presence Detection

Presence detection algorithms determine whether a targeted LDA device is present within the CXR imaging field without identifying its precise location or assessing whether it is appropriately positioned. As the simplest form of automated LDA analysis, presence detection can serve as an initial screening step before more computationally intensive localization or position classification algorithms are applied. Based on our review, Convolutional Neural Network (CNN) architectures serve as the primary backbone for LDA presence detection. Several established CNN architectures, including GoogLeNet, ResNet, and EfficientNet variants, have demonstrated high performance for detecting a variety of devices, including CVCs, ETTs, thoracic drains, NGTs, and UACs [11,14,38,39,40]. Beyond these established architectures, specialized AI models have also been developed, including hybrid DenseNet121 and DeepLabV3+ frameworks [17,28], multi-stage cascaded CNNs [25], unified bounding-box detection frameworks [41], and specialized Scale-Recurrent Networks adapted for thin pediatric catheters [42].

For presence detection, image-level CNN classifiers may perform well because this task primarily requires recognition of global and local visual features indicating whether a device is present, without requiring precise delineation of its trajectory or tip. Residual and densely connected architectures may further facilitate feature reuse and gradient propagation, which can be advantageous for detecting thin, low-contrast devices against complex thoracic anatomy.

Importantly, AI systems are capable of distinguishing among different categories of LDA devices when trained using multi-class or multi-label frameworks. Such models can identify multiple device types, including ETTs, enteric tubes, and vascular catheters, within the same CXR; however, many currently published algorithms remain optimized for a single device category, and performance may vary when multiple overlapping devices are present.

### 4.2. Device Localization

Device localization algorithms localize a targeted LDA device within the image by delineating its full course, estimating the location of its distal tip, or both. Localization is clinically valuable for visualizing device position and serves as the foundation for position classification. For some devices, such as tracheostomy tubes, localization alone may provide sufficient clinical information because malposition is relatively uncommon.

Semantic segmentation models are commonly used to generate pixel-level mappings of the continuous trajectory of the LDA devices and provide visual interpretability for clinicians. Based on our review, U-Net and its variants are the most widely used models for semantic segmentation of LDAs. U-Net and U-Net++ have been applied to generate segmentation masks for ETTs, CVCs, and PICCs [26,43,44,45,46,47]. The self-configuring nnU-Net, which automates preprocessing and network topology, has been successfully applied to map NGTs and Right IJ Lines [34,48,49,50]. DeepLabV3+ has also been used to segment narrow devices, such as NGTs and ETTs, by leveraging its Atrous Spatial Pyramid Pooling (ASPP) module. The ASPP module enables the network to capture multi-scale contextual information without losing spatial resolution, which is critical for preserving the fine structural details of thin LDAs [17,28,51].

For many LDAs, accurate localization of the distal tip is more clinically relevant than segmentation of the entire device, as placement appropriateness is ultimately determined by the relationship between the tip and adjacent anatomical landmarks (e.g., carina, CAJ, or thoracic inlet). Tip localization can be achieved by post-processing segmentation masks to identify the most distal end point along the segmented device. Commonly used models for this segmentation-based approach include DeepLabV3+ [17,28,51], nnU-Net [34], or standard U-Net architectures [26,42,44,52]. Alternatively, object detection algorithms and coordinate regression models can localize the coordinates of device tips directly without requiring full trajectory segmentation. Object detection frameworks, such as YOLO variants (YOLOv3, YOLOv5) [37,41], Faster R-CNN [47,53], Fully Convolutional One-Stage Object Detection (FCOS) [54], or RetinaNet [55], project bounding boxes directly over the device tip. To achieve precise, single-pixel localization, coordinate regression networks, such as Cascaded CNNs [25] and modified DenseNet121 [56], estimate the pixel coordinates of device tips through direct numerical coordinate regression or 2D Gaussian probability heatmaps.

These architectural differences reflect the level of spatial information required by the localization task. Encoder–decoder segmentation networks are well suited to tracing the continuous trajectories of thin LDAs because skip connections help preserve fine spatial features while incorporating broader contextual information. When the clinical objective is limited to distal tip localization, object-detection, coordinate-regression, or heatmap-based approaches can instead target a localized region or point directly without requiring reconstruction of the entire device course.

### 4.3. Position Classification

Position classification algorithms determine whether a targeted LDA is appropriately positioned relative to key anatomical landmarks. Rather than simply identifying where an LDA device is located, position classification interprets that information against established clinical placement criteria to reach a determination of device position. Position classification algorithms generally follow two methodological paradigms: image-level classification and localization-based classification.

The first paradigm uses end-to-end CNN classifiers trained on image-level labels to directly classify device position as satisfactory or malpositioned without requiring an explicit distal tip localization output. Standard CNN-based classifiers, including Inception V3 [19,27,57], DenseNet121 [58], GoogLeNet [40], and VGG16 with tensor projection layers [59], have demonstrated high performance for image-level position classification using global image features. Additionally, multi-label frameworks [3] and multitask CNNs [32] have been developed to classify multiple placement attributes simultaneously [11].

The second paradigm, localization-based classification, builds on device localization by combining segmentation or tip localization with anatomical landmark detection to directly evaluate placement criteria. This is frequently achieved via dual-stage pipelines that combine a segmentation framework (e.g., nnU-Net, DeepLabV3+, or U-Net++) with a classification backbone (e.g., ResNet-50, DenseNet-121) [17,28,46,48,49,50,51,52]. Alternatively, unified localization-based frameworks using YOLOv3, YOLOv5, Faster R-CNN, U-Net, and RetinaNet [37,41,43,53,55,56] have been used to explicitly measure distances relative to anatomical landmarks. Several commercial AI tools implementing these approaches have been deployed in ICUs to assist with automated assessment of critical device positions [13,20].

The relative advantage of these approaches likely depends on the clinical task. End-to-end classifiers can exploit global image context and require less detailed annotation, but may rely on indirect imaging features rather than explicitly identifying the device–anatomy relationship. Localization-based or dual-stage approaches may therefore be advantageous when correct positioning is defined by measurable spatial relationships between the device tip and anatomical landmarks, although errors in the localization stage can propagate to the final classification.

## 5. Quantitative Performance of AI Models

This section summarizes the quantitative performance of AI models for the four primary categories of LDA devices. Summary tables for each device category are provided in Table 1, Table 2, Table 3 and Table 4.

### 5.1. Artificial Airway Models

Deep learning models consistently demonstrate excellent performance for automated detection of ETTs across both pediatric and adult populations (Table 1). In pediatric and neonatal ICU populations, ResNet-based architectures achieved an average precision of 0.989 for ETT presence detection [38]. Similar performance has been reported in adult populations, where AUROC values range from 0.989 using pre-trained GoogLeNet [40] to 1.0 utilizing DenseNet121, DeepLabV3+, and commercial algorithms [13,17,39]. High detection performance has also been maintained in multi-label frameworks, with YOLO-v3 demonstrating a sensitivity of 99.4% and specificity of 99.2% [41].

In addition to presence detection, numerous studies have evaluated automated localization of ETTs through segmentation of the tube trajectory, identification of the distal tip, and localization of key anatomical landmarks (e.g., carina). Models for trajectory segmentation have consistently demonstrated high performance across both pediatric and adult populations. In pediatric cohorts, Scale-Recurrent Networks achieved an F-beta measure of 0.80 for ETT segmentation on neonatal radiographs despite being trained exclusively on adult data [42]. In adult cohorts, standard U-Net architectures achieved a Dice coefficient of 87.3% for ETT segmentation and an absolute ETT-to-carina measurement difference of 0.48 to 0.60 cm, closely mirroring the inter-reader variability of human radiologists [26]. Advanced architectures such as U-Net++ achieved Dice scores of 0.893 for the ETT and 0.841 for the trachea, with an Intersection over Union (IoU) of 0.8683 and average distance errors of 2.9 mm [44,46]. Similarly, DeepLabV3+ achieved a Dice score of 0.854 and an overall localization error of 1.1 cm [51].

Because clinical placement depends primarily on the position of the distal tip, several investigators have focused specifically on tip localization. Two-stage Mask R-CNN, DenseNet121, and multitask CNNs achieved localization errors ranging from 2.6 to 3.04 mm, comparable to those of human experts [52,53,56]. Among these approaches, Mask R-CNN demonstrated superior localization precision compared with 11 clinicians [53], while multitask CNNs reported tip localization errors of less than 3 mm in more than 86% of evaluated ETTs [52]. Additional architectures, including FCOS, RetinaNet, Cascaded CNNs, and YOLO-v3, achieved tip localization errors ranging between 3.73 and 8.2 mm, demonstrating meaningful variability in localization performance across studies [24,25,41,54,55]. This between-study variability likely reflects multiple methodological factors rather than model architecture alone. Differences in patient populations, image acquisition protocols and spatial resolution, device visibility, and annotation procedures can influence distal tip localization accuracy. Model design may also contribute, as segmentation-based approaches infer the distal tip from the segmented device trajectory, while object-detection and regression-based approaches localize the tip more directly. In addition, differences in the definition of the localization target and reference standard may further limit direct comparison of localization errors across studies.

Performance for automated ETT position classification is more variable compared to presence detection or device localization (Table 1). Standard image-level classification models, including VGG16 with ROI-cropped images and commercial multi-label frameworks, achieved AUROCs ranging from 0.86 to 0.92 for classifying satisfactory versus malpositioned ETTs [39,59].

Other architectures, including DenseNet121 and GoogLeNet, reported lower generalized malposition AUROCs ranging from 0.734 to 0.847 [17,40,51]. For more granular position classification, U-Net++ combined with EfficientNet-b0 achieved an accuracy of 0.922 across four ETT depth categories [46], while Conv-MTD achieved an AUROC of 0.99 for three-class ETT placement classification [11].

Performance generally improves when models are designed to identify clinically significant malpositions rather than all forms of malposition. Unified classification architectures and multitask CNNs reported AUROCs exceeding 0.98 for detecting critical ETT malpositions [52], with sensitivities ranging from 93.9% to 100% and specificities from 96.7% to 100% [13,27]. AI assistance improves clinician performance. Commercial AI increased clinician accuracy for detecting critical ETT malpositions from 79.3% to 89.0% [20], while RetinaNet improved human sensitivity for detecting dangerously low ETTs from 0.76 to 0.91 [55].

### 5.2. Enteric Tube Models

Automated detection of enteric tubes has emerged as one of the most active applications of AI-assisted assessment (Table 2). Across both pediatric and adult populations, AI models consistently demonstrate excellent performance for enteric tube detection. ResNet-50 architectures successfully identified the presence of NGTs in a pediatric cohort with an average precision of 0.977 [38]. The high detection accuracy extends to the general adult population, with standard ResNet-50, deep convolutional neural networks (DCNNs), and DeepLabV3+ algorithms predicting the presence of enteric tubes with AUC scores ranging from 0.98 to 0.998 [14,28,39].

For device localization, algorithms demonstrate varied performance in segmenting the continuous trajectory and localizing the distal tip of enteric tubes. In pediatric applications, Scale-Recurrent Networks successfully adapted adult training data to neonatal cohorts with an F-beta score of 0.80 while concurrently segmenting the ETT, NGTs, and vascular lines [42]. In adult populations, unified models based on nnU-Net and ResNet backbones achieved a Dice coefficient of 86.56% for NGT segmentation on internal datasets, which decreased to 79.37% on external validation data [48]. Several other studies using nnU-Net, ResNet, and DeepLabV3+ achieved lower Dice scores ranging from 0.646 to 0.665 [28,49,50]. Relatively few studies have reported quantitative performance for distal tip localization.

DeepLabV3+ and DenseNet121 achieved mean tip localization errors ranging from 1.64 to 2.83 cm [28,51]. The larger distal tip localization error reported for enteric tubes compared with ETTs may partly reflect the greater difficulty in visualizing and tracing the distal NGT. On portable supine CXRs, variable image quality, overlap with mediastinal structures and other support devices, and looping or aberrant NGT trajectories can reduce tube visibility and complicate localization. Wang et al. [28] also noted that the portion of the NGT near the distal tip was particularly challenging to segment. However, direct comparison with ETT studies should be interpreted cautiously because the studies used different datasets, model architectures, and evaluation protocols.

Assessing enteric tube placement yields more variable performance than simply detecting tube presence, with outcomes varying by classification task. For general NGT misplacement classification, neural network ensembles and nnU-Net models achieved AUROCs ranging from 0.77 to 0.848 [19,48]. Higher performance was achieved by dual-stage frameworks that combined localization with position classification, reporting AUROCs between 0.964 and 1 for NGT placement evaluation [28,49,50,51]. When evaluating a broader range of enteric tubes (double-lumen, fine-bore, NJT, and NDT) using diverse datasets, such as the Catheter and Line Position (CLiP) dataset, models achieved general malposition AUROCs ranging from 0.839 to 0.89, and satisfactory placement AUROCs up to 0.91 [21,28]. Multi-label frameworks achieved excellent discrimination for classifying enteric tube placement into normal (AUROC = 0.98), borderline (AUROC = 0.97), and abnormal (AUROC = 0.96) placements [11]. Performance generally improved when models were designed to identify severe or clinically significant malpositions, with DenseNet121-based models and neural network ensembles reporting AUROCs between 0.87 and 0.98 [19,28,57,58].

### 5.3. Central Venous Catheter Models

AI models demonstrate excellent performance for automated detection of CVCs across both pediatric and adult populations (Table 3). In highly vulnerable pediatric and neonatal populations, ResNet-50-based architectures have demonstrated excellent performance for detecting specialized neonatal vascular lines, including UACs and UVCs, achieving average precisions of 0.979 and 0.937, respectively [38]. In adult cohorts, ResNet-50 and commercial models such as Annalise CXR exhibit excellent discriminative capability, reporting AUROCs ranging from 0.90 to greater than 0.99 for CVC detection [14,39].

For device localization, studies have evaluated both segmentation of catheter trajectories and localization of distal catheter tips. In neonatal populations, the Scale-Recurrent Network accurately traced UAC and UVC trajectories, achieving an F-beta score of 0.80 for the combined catheter class [42]. More recently, topology-preserving embedded networks have been developed to address the challenges of low-contrast pediatric imaging and demonstrated highly precise PICC localization, with mean tip distance errors below 3.125 mm [33]. In adult CVC segmentation applications, a model combining U-Net++ and EfficientNet-B1 achieved a Dice score of 0.84 [43]. Similarly, nnU-Net models achieved an average symmetric surface distance of 1.41 mm for segmenting right IJ lines and a mean tip localization error of 8.29 mm [34]. For PICC segmentation, a multi-stage deep learning framework achieved a Dice score of 0.92 and an internal tip RMSE of 9.77 mm [18], while alternative multi-task frameworks integrating U-Net and Faster R-CNN reported more modest segmentation performance (Dice = 0.58) [47]. Direct tip localization approaches have generally achieved sub-centimeter accuracy. Cascade FCN yielded a mean absolute tip distance error of 3.10 mm (RMSE = 3.71 mm) [35], whereas multi-task CNNs localized catheter tips within 3 mm of the reference standard in 77% of cases [52]. The variability in reported tip localization performance likely reflects differences in catheter type, patient population, image quality, annotation strategy, and localization methodology, as well as differences between internal and external validation datasets.

Performance for automated CVC position classification remains consistently high across multiple AI architectures. A combined architecture incorporating U-Net++ and EfficientNet-B1 achieved an AUROC of 0.99 for identifying correctly positioned CVCs and an AUROC of 0.95 for detecting malpositioned catheters [43]. Similarly, multi-task CNNs reported AUROC values exceeding 0.93 for overall CVC malposition classification [52], while commercial algorithms demonstrated slightly lower performance, with an AUROC of 0.87 for detecting unsatisfactory CVC placement [39]. For multi-class classification tasks, Jung et al. [45] employed a U-Net++ and EfficientNet-B4 framework to classify CVC placements as shallow, appropriate, or deep, achieving an overall accuracy of 0.82 and an F1-score of 0.73. Likewise, the Conv-MTD framework stratified catheter position into normal, borderline, and abnormal categories, yielding AUROC values of 0.90, 0.84, and 0.90, respectively [11].

### 5.4. Drain Models

AI models demonstrate excellent performance for automated detection of thoracic drainage devices (Table 4). ResNet-50 models successfully identified thoracic drains on CXRs with an AUC of 0.99, demonstrating diagnostic accuracy comparable to expert radiologists [14].

For device localization, published studies have primarily focused on device localization using object detection frameworks rather than segmentation-based approaches. Using the YOLOv5 architecture, Kim et al. localized chest PCDs on CXRs, achieving a mean average precision at 50% intersection over union (mAP50) of 0.86 [37].

Performance for automated drain position classification has also been encouraging. By analyzing localized bounding boxes, a YOLOv5 algorithm differentiated appropriately positioned from malpositioned chest PCDs with an overall accuracy of 0.88, sensitivity of 0.86, and specificity of 0.92. Notably, prediction of drain malfunction associated with malposition achieved an even higher sensitivity of 0.93 and specificity of 0.95, demonstrating the potential of AI to support both radiologists and clinicians in assessing thoracic drains [37].

## 6. Discussion

AI models have achieved consistently high performance for automated assessment of LDAs on CXRs, although performance varies substantially according to the underlying task. Across the four LDA device categories, including artificial airways, enteric tubes, CVCs, and drains, a consistent pattern emerged regarding algorithm performance. Presence detection achieved the highest and most reproducible diagnostic accuracy, with several ETT detection models reporting AUROCs near 0.99 or higher across both adult and pediatric populations [14,38,39]. Device localization also demonstrated strong performance, although performance varied with device complexity, anatomical variability, and image quality. In contrast, position classification remains the most challenging task because it requires not only accurate device localization but also precise detection of relevant anatomical landmarks before a clinically meaningful decision can be made [42]. This progressive increase in task complexity was consistently reflected by declining performance across the reviewed studies, suggesting that future methodological advances should focus increasingly on anatomically informed position classification rather than further improvements in device detection alone.

While a limited number of models have demonstrated performance approaching that of expert radiologists, the majority have yet to achieve the robustness and generalizability required for routine clinical deployment. Therefore, the current role of AI is primarily to support rather than replace clinical expertise [13,19,20]. In this role, AI is particularly valuable for bedside CXRs, where it may help clinicians identify potentially life-threatening malpositions before a formal radiology report becomes available [17,57,58]. Collectively, these findings suggest that AI is best positioned as a CDS tool that improves workflow efficiency, enhances diagnostic consistency, and strengthens patient safety [14,20,39].

One important finding of this review is the scarcity of pediatric and neonatal evidence despite the substantial clinical need within these populations. Although several studies demonstrated excellent performance for detecting and localizing neonatal ETTs, umbilical catheters, and enteric feeding tubes [38,42], pediatric-specific investigations remain limited. Most currently available algorithms were developed using adult datasets [33,42]. This discrepancy is clinically significant because pediatric CXRs present unique imaging challenges, including substantially smaller anatomical structures, lower image contrast resulting from radiation dose reduction strategies, and the frequent coexistence of multiple overlapping life-support devices within a restricted field of view [33,38,60,61]. Moreover, even minimal deviations in device position may result in serious complications in neonates and young children [56]. Consequently, future research should prioritize pediatric-specific datasets and model architectures capable of accommodating substantial scale variation.

Despite the encouraging progress demonstrated across literature, several methodological limitations continue to impede broader clinical implementation. Terminology and thresholds for device malposition vary across device types and individual studies. In general, satisfactory positioning refers to placement within the accepted device-specific anatomical target range, whereas borderline or suboptimal positioning describes deviation from the ideal position that may warrant reassessment or adjustment but does not necessarily represent an immediate high-risk situation. Clinically significant malposition refers to a position associated with substantial risk of impaired device function or patient harm and generally requiring prompt clinical attention or correction. Some studies further use terms such as severe or critical malposition to describe specific high-risk subsets; these terms and their quantitative thresholds are study-specific and are therefore retained as originally defined when individual study results are reported. Representative clinically significant malpositions include endobronchial ETT placement, enteric tube entry into the respiratory tract, excessive advancement of a central venous catheter into the heart or another unintended vessel, and displacement of a drainage catheter that compromises drainage. From a data perspective, severe class imbalance remains a persistent challenge because clinically significant device malposition occurs far less frequently than correctly positioned devices [11,48,49,58]. Consequently, many algorithms are trained predominantly on normal examinations and may exhibit reduced sensitivity for detecting rare, but clinically significant device malposition. In addition, the lack of standardized annotation protocols and device-specific labeling may reduce both model performance and the comparability of published studies. A representative example is the publicly available CLiP dataset, in which multiple enteric feeding devices, including nasogastric, nasoduodenal, and nasojejunal tubes, are collectively labeled as nasogastric tubes [11,28,39]. Although these devices share similar proximal courses through the esophagus, they differ substantially in their distal trajectories and intended anatomical endpoints [28]. Consequently, models trained or externally validated on such datasets may experience reduced performance, particularly for malposition assessment, because the tube tip is the primary determinant of appropriate placement [28]. More granular annotation of individual enteric tube types, their distal tips, and their corresponding placement criteria would therefore improve model development, facilitate fair comparison across studies, and enhance the clinical specificity of AI-assisted device assessment [28,39]. Furthermore, the reference standard itself is inherently imperfect because annotation of catheter tips, airway landmarks, and other anatomical reference points is subject to inter-observer variability among experienced radiologists [14,26,52].

Future research should therefore focus on translating these promising algorithms into robust clinical tools. Rather than further improving image-level classification alone, future efforts should continue emphasizing anatomically informed localization frameworks that integrate device segmentation, landmark identification, and quantitative distance measurements into clinically interpretable decision-making pipelines [34,43,48,52]. The development of unified multi-device algorithms capable of simultaneously assessing the wide array of medical devices encountered on bedside CXRs would further enhance their clinical utility [11,14,38]. Prospective multicenter validation across heterogeneous adult and pediatric populations will be essential to establish model robustness, generalizability, and clinical effectiveness under real-world conditions [19,33,39,49].

Even highly accurate algorithms will require careful integration into radiology and bedside workflows before they can achieve widespread adoption. AI systems for LDA assessment must be evaluated not only by conventional metrics, such as AUROC, Dice, and localization error, but also by their impact on report turnaround time, urgent case prioritization, false-positive alert burden, clinician trust, and downstream patient outcomes. In addition, model calibration, uncertainty estimation, failure-mode analysis, regulatory clearance, and post-deployment performance monitoring will be essential to ensure safe and reliable use across institutions, imaging equipment, patient populations, and clinical settings. Together, these advances may facilitate the translation of AI-assisted LDA assessment from retrospective algorithm development into routine clinical practice, improving workflow efficiency, enhancing diagnostic consistency, and ultimately contributing to safer patient care.

## 7. Conclusions

AI has emerged as a powerful tool for the automated assessment of LDAs on CXRs, demonstrating substantial progress across a wide range of clinical applications. Among the reviewed studies, AI models consistently achieved the highest performance for presence detection, followed by device localization, whereas position classification remained the most challenging task because it required integration of device position with clinically relevant anatomical landmarks. Collectively, these findings demonstrate the growing potential of AI to improve bedside imaging workflows through rapid, consistent, and reproducible assessment of medical devices. However, the evidence supporting these findings is derived predominantly from adult populations, and pediatric- and neonatal-specific validation remains limited.

Despite these advances, several important challenges remain before widespread clinical implementation can be achieved. Current evidence suggests that AI is best positioned to function as a CDS tool that complements, rather than replaces, radiologist expertise. With continued methodological improvements, prospective multicenter validation, and thoughtful integration into radiology and bedside workflows, AI-assisted LDA assessment has the potential to become an important component of routine clinical practice, improving diagnostic efficiency, reducing interpretive variability, and ultimately contributing to safer patient care.

## Figures and Tables

**Table 1:** Summary of AI models for airway assessment.

Reference	Dataset & Sample Size	Age Group	AI Architecture	Presence Detection	Device Localization: Trajectory Segmentation	Device Localization: Distal Tip Localization	Position Classification
Henderson et al., 2021 [38]	Royal Univ. PACS; *n* = 777	Neonates	ResNet-50	AP: 0.989	—	—	—
Yi et al., 2020 [42]	Open-i (NIH) & Local; *n* = 2550	Train: Adults; Test: Neonates (<4 wk)	Scale-Recurrent Network	—	F-beta: 0.800	—	—
Abbas et al., 2026 [11]	RANZCR CLiP; *n* = 30,083	Adults	Conv-MTD	—	—	—	AUROC: Normal 0.990, Borderline 0.955, Abnormal 0.950
Wang et al., 2025 [28]	NTUH-20 & NTUH-YB; *n* = 7378	Adults (≥20 yr)	DeepLabV3+	AUROC: 0.994–1.000	Dice: 0.839 (NTUH-YB), 0.854 (NTUH-20)	ETT MAE: 11.0 mm	AUROC: 0.734–0.847
Novak et al., 2025 [20]	CRIS database; *n* = 400	Adults (≥18 yr)	GEHC Critical Care Suite	—	—	—	Human accuracy gain: Overall 0.774 (from 0.736), Critical 0.890 (from 0.793)
Rueckel et al., 2024 [52]	Multi-center (>10 sites); *n* = 9889	Adults (≥21 yr)	Multitask CNN	—	—	—	AUROC: 0.980
An et al., 2024 [13]	Seoul National Univ.; *n* = 1722	Adults (≥19 yr)	Lunit INSIGHT CXR	AUROC: 1.000	—	ETT-Carina MAE: 5.1–6.2 mm	Sens: 1.000; Spec: 0.967–1.000 (Critical position)
Tang et al., 2023 [39]	Aus. Private & MIMIC-CXR; *n* = 4568	Adults (≥16 yr)	Annalise CXR v2.0	AUROC: 0.990	—	—	AUROC: 0.860–0.910
Hsu et al., 2023 [44]	Dalin Tzu Chi & RANZCR; *n* = 1455	Adults	RegNet encoder, U-Net++ decoder	—	IoU: 0.868	ETT MAE: 2.9 mm	—
Brown et al., 2023 [24]	Medical & Surgical ICUs; *n* = 2000	Adults (≥18 yr)	Semantically Embedded DNN	—	—	ETT MAE: 7.2 mm	PPV: 0.830; NPV: 0.980
Oliver et al., 2023 [55]	RadioICU & MIMIC-CXR; *n* = 2090	—	CarinaNet	—	—	ETT MAE: 5.1 mm	Human sensitivity gain: 0.910 (from 0.760)
Huang et al., 2022 [53]	National Cheng Kung Univ.; *n* = 2058	Adults	Mask R-CNN	—	—	Median error: ETT 2.8–3.0 mm	Sens: 0.721–0.773; Spec: 0.952–0.953
Jung et al., 2022 [46]	Hanyang Univ. ER; *n* = 1985	Adults (63.5 ± 18.3 yr)	U-Net++, EfficientNet-B0	—	Dice: ETT 0.893, Trachea 0.841	—	4-label accuracy: 0.922
Mao et al., 2022 [54]	National Cheng Kung Univ.; *n* = 2020	Adults	FCOS + Coarse-to-Fine Attention	—	—	MAE: 3.7–4.3 mm; ETT-Carina MAE: 5.3 mm	Classification accuracy: 0.889
Tsai et al., 2022 [56]	Taipei Medical Univ.; *n* = 390	Adults	DenseNet-121 encoder, CNN decoder	—	—	ETT MAE: 3.0 mm	AUROC: 0.938
Schultheis & Lakhani, 2022 [26]	Thomas Jefferson Univ.; *n* = 826	Adults (>18 yr)	U-Net	—	Dice: 0.873	ETT-Carina MAE: 4.8–6.0 mm	—
Yuan et al., 2021 [59]	Blinded PACS; *n* = 4293	Adults	VGG-16	—	—	—	AUROC: 0.920
Harris et al., 2021 [41]	vRad; *n* = 49,112	Adults (56.8–60.0 yr)	YOLOv3	Sens: 0.994; Spec: 0.992	—	MAE: 7.0 mm	Sens: 0.759; Spec: 0.885
Kara et al., 2021 [25]	MIMIC-CXR; *n* = 16,000	Adults	Cascaded CNN	AUROC: 0.996	—	ETT MAE: 8.2 mm	—
Niehues et al., 2021 [14]	Charité Level 1 Center; *n* = 18,361	Adults (62.6 ± 16.7 yr)	ResNet-50	AUROC: 0.990	—	—	—
Lakhani et al., 2021 [27]	Thomas Jefferson Univ.; *n* = 22,960	Adults (≥18 yr)	Inception-v3	—	—	—	Sens: 0.939; Spec: 0.977
Lakhani et al., 2017 [40]	Local PACS; *n* = 600	Adults	GoogLeNet	AUROC: 0.989	—	—	AUROC: 0.809

Table Footnotes & Abbreviations. AI & Architectures: CNN = Convolutional Neural Network; DNN = Deep Neural Network; FCOS = Fully Convolutional One-Stage Object Detector; MTD = Multi-Task Deep Learning; VGG = Visual Geometry Group; YOLO = You Only Look Once. Performance Metrics: AP = Average Precision; AUROC = Area Under the Receiver Operating Characteristic Curve; IoU = Intersection over Union; MAE = Mean Absolute Error; NPV = Negative Predictive Value; PPV = Positive Predictive Value; Sens = Sensitivity; Spec = Specificity. Clinical Terms & Datasets: CXR = Chest Radiograph; ER = Emergency Room; ETT = Endotracheal Tube; NTUH = National Taiwan University Hospital; ICU = Intensive Care Unit; MIMIC-CXR = Medical Information Mart for Intensive Care Chest X-Ray; NIH = National Institutes of Health; PACS = Picture Archiving and Communication System; vRad = Virtual Radiologic; RANZCR CLiP = Royal Australian and New Zealand College of Radiologists Comprehensive List of Presentations.

**Table 2:** Summary of AI models for enteric tube assessment.

Reference	Dataset & Sample Size	Age Group	AI Architecture	Presence Detection	Device Localization: Trajectory Segmentation	Device Localization: Distal Tip Localization	Position Classification
Henderson et al., 2021 [38]	Royal Univ. PACS; *n* = 777	Neonates	ResNet-50	AP: 0.977	—	—	—
Yi et al., 2020 [42]	Open-i (NIH) & Local; *n* = 2550	Train: Adults; Test: Neonates (<4 wk)	Scale-Recurrent Network	—	F-beta: 0.800	—	—
Abbas et al., 2026 [11]	RANZCR CLiP; *n* = 30,083	Adults	Conv-MTD	—	—	—	AUROC: Normal 0.980, Borderline 0.970, Abnormal 0.960
Moon et al., 2026 [48]	Internal & MIMIC-CXR; *n* = 7028	Adults	Unified Model	—	Dice: 0.866 (Internal), 0.794 (External)	—	AUROC: 0.972 (Internal), 0.848 (External)
Park et al., 2025 [49]	Kangwon National Univ.; *n* = 135	Adults (77.3–80.7 yr)	Dual-Stage	—	Dice: 0.654	—	AUROC: 0.970; Sens: 0.961; Spec: 0.857
Wang et al., 2025 [28]	NTUH-20 & NTUH-YB; *n* = 7378	Adults (≥20 yr)	DeepLabV3+	—	Dice: 0.646 (NTUH-YB), 0.665 (NTUH-20)	NGT MAE: 16.4–28.3 mm	AUROC: 0.964
Park et al., 2025 [50]	Hallym, Gangneung, Kangwon; *n* = 2627	Adults (77.5 ± 11.2 yr)	Dual-Stage	—	Dice: 0.654	—	AUROC: 1.000 (Internal), 0.923 (External)
Tang et al., 2023 [39]	Aus. Private & MIMIC-CXR; *n* = 4568	Adults (≥16 yr)	Annalise CXR v2.0	AUROC: 0.990	—	—	AUROC: 0.890–0.900
Drozdov et al., 2023 [19]	NHS Greater Glasgow & Clyde; *n* = 7416	Adults (≥16 yr)	Neural Network Ensemble	—	—	—	AUROC: 0.820 (Satisfactory), 0.770 (Malpositioned); Human accuracy gain: 0.780 (from 0.690)
Mallon et al., 2022 [58]	Imperial College Healthcare; *n* = 6207	Adults (>18 yr)	DenseNet-121	—	—	—	AUROC: 0.920; Sens: 0.800; Spec: 0.920
Niehues et al., 2021 [14]	Charité Level-1 Center; *n* = 18,361	Adults (62.6 ± 16.7 yr)	ResNet-50	AUROC: 0.980	—	—	—
Singh et al., 2019 [57]	Blinded PACS; *n* = 5475	—	Inception-v3	—	—	—	AUROC: 0.870

Table Footnotes & Abbreviations. AI & Architectures: MTD = Multi-Task Deep Learning; Performance Metrics: AP = Average Precision; AUROC = Area Under the Receiver Operating Characteristic Curve; MAE = Mean Absolute Error; Sens = Sensitivity; Spec = Specificity. Clinical Terms & Datasets: CXR = Chest Radiograph; MIMIC-CXR = Medical Information Mart for Intensive Care Chest X-Ray; NGT = Nasogastric Tube; NIH = National Institutes of Health; NHS = National Health Service; NTUH = National Taiwan University Hospital; PACS = Picture Archiving and Communication System; RANZCR CLiP = Royal Australian and New Zealand College of Radiologists Comprehensive List of Presentations.

**Table 3:** Summary of AI models for central venous catheter (CVC) assessment.

Reference	Dataset & Sample Size	Age Group	AI Architecture	Presence Detection	Device Localization: Trajectory Segmentation	Device Localization: Distal Tip Localization	Position Classification
Yin et al., 2026 [33]	Multi-center (3 centers); *n* = 1184	Pediatric and Adult	TopNet	—	—	PICC MAE: 3.125 mm	—
Henderson et al., 2021 [38]	Royal Univ. PACS; *n* = 777	Neonates	ResNet-50	AP: UAC 0.979, UVC 0.937	—	—	—
Yi et al., 2020 [42]	Open-i (NIH) & Local; *n* = 2550	Train: Adults; Test: Neonates (<4 wk)	Scale-Recurrent Network	—	F-beta: 0.800	—	—
Abbas et al., 2026 [11]	RANZCR CLiP; *n* = 30,083	Adults	Conv-MTD	—	—	—	AUROC: Normal 0.900, Borderline 0.840, Abnormal 0.900
Stroeder et al., 2025 [43]	RANZCR CLiP & In-house; *n* = 31,089	Adults (69 ± 7 yr)	U-Net++, EfficientNet-B1	—	Dice: 0.840; Hausdorff: 1.35 mm	—	AUROC: 0.990 (Correct), 0.950 (Incorrect)
Wei et al., 2025 [34]	UCLA Health (In-house); *n* = 937	ICU Patients	nnU-Net	—	RIJL ASSD: 1.41 mm	RIJL Tip MAE: 8.29 mm	—
Rueckel et al., 2024 [52]	Multi-center (>10 sites); *n* = 9889	Adults (≥21 yr)	Multitask CNN	—	—	—	AUROC: 0.930
Park et al., 2023 [18]	Samsung Medical & RANZCR; *n* = 1250	Not reported	Multi-Fragment CNN	—	Dice: 0.920 (Internal)	PICC RMSE: 9.77 mm (Internal), 56.71 mm (External)	—
Tang et al., 2023 [39]	Aus. Private & MIMIC-CXR; *n* = 4568	Adults (≥16 yr)	Annalise CXR v2.0	AUROC: 0.990	—	—	AUROC: 0.870
Jung et al., 2022 [45]	Hanyang Univ. ER; *n* = 808	Adults (18–80 yr)	U-Net++, EfficientNet-B4	—	—	—	3-label accuracy: 0.820; F1-score: 0.730
Niehues et al., 2021 [14]	Charité Level-1 Center; *n* = 18,361	Adults (62.6 ± 16.7 yr)	ResNet-50	AUROC: 0.990	—	—	—
Yu et al., 2020 [47]	Quhua & Second Affiliated; *n* = 348	—	U-Net and Faster R-CNN	—	PICC F1-score: 0.580	Tip detection F1-score: 0.740	—
Lee et al., 2018 [35]	Massachusetts General; *n* = 550	—	Cascade FCNs	—	—	PICC Tip MAE: 3.10 mm	—

Table Footnotes & Abbreviations. AI & Architectures: CNN = Convolutional Neural Network; FCN = Fully Convolutional Network; MTD = Multi-Task Deep Learning; nnU-Net = No-New-Net (Self-configuring U-Net); R-CNN = Region-Based Convolutional Neural Network. Performance Metrics: AP = Average Precision; ASSD = Average Symmetric Surface Distance; AUROC = Area Under the Receiver Operating Characteristic Curve; MAE = Mean Absolute Error; RMSE = Root Mean Square Error. Clinical Terms & Datasets: CXR = Chest Radiograph; ER = Emergency Room; ICU = Intensive Care Unit; MIMIC-CXR = Medical Information Mart for Intensive Care Chest X-Ray; NIH = National Institutes of Health; PACS = Picture Archiving and Communication System; PICC = Peripherally Inserted Central Catheter; RIJL = Right Internal Jugular Line; UAC = Umbilical Arterial Catheter; UVC = Umbilical Venous Catheter; RANZCR CLiP = Royal Australian and New Zealand College of Radiologists Comprehensive List of Presentations.

**Table 4:** Summary of AI models for thoracic drain assessment.

Reference	Dataset & Sample Size	Age Group	AI Architecture	Presence Detection	Device Localization: Trajectory Segmentation	Device Localization: Distal Tip Localization	Position Classification
Niehues et al., 2021 [14]	Charité Level-1 Center; *n* = 18,361	Adults (62.6 ± 16.7 yr)	ResNet-50	AUROC: 0.990	—	—	—
Kim et al., 2024 [37]	Jeju National Univ. Hospital; *n* = 1217	Adults (71.6 ± 0.4 yr)	YOLOv5	—	—	mAP50: 0.860	Sens: 0.860; Spec: 0.920; Accuracy: 0.880 (Malpositioned PCDs)

Table Footnotes & Abbreviations. AI & Architectures: YOLO = You Only Look Once. Performance Metrics: AUROC = Area Under the Receiver Operating Characteristic Curve; mAP50 = Mean Average Precision at IoU threshold of 0.50; Sens = Sensitivity; Spec = Specificity. Clinical Terms & Datasets: PCD = Percutaneous Chest Drain.

## Data Availability

No new data were created or analyzed in this study. Data sharing is not applicable to this article.
